# *Trypanosoma evansi* in Brazil: first evidence of infection in equines from Northeast region

**DOI:** 10.1590/S1984-29612025032

**Published:** 2025-06-20

**Authors:** Jordeano Araujo Sousa, Leandro Macedo Miranda, Danielle Jordany Barros Coutinho, Thaliane França Costa, Sidilene Pereira Costa, Úrsula Silva Freitas, Francisco Borges Costa, Rosangela Zacarias Machado, Rita de Maria Seabra Nogueira, Andréa Pereira da Costa

**Affiliations:** 1 Laboratório de Parasitologia e Doenças Parasitárias, Universidade Estadual do Maranhão – UEMA, São Luís, MA, Brasil; 2 Vector-Borne Bioagents Laboratory, Departamento de Patologia, Reprodução e Saúde Única, Faculdade de Ciências Agrárias e Veterinárias, Universidade Estadual Paulista – UNESP, Jaboticabal, SP, Brasil

**Keywords:** Equine protozoan infections, equine health, Trypanosomosis, Infecções por protozoários equinos, saúde equina, Tripanosomose

## Abstract

The hemoprotozoan *Trypanosoma evansi* is a parasite that infects mammals, causing an infection known as trypanosomiasis. There is no report of *T. evansi* in horses in the State of Maranhão, highlighting the need to assess exposure and infection by the parasite and generate data for its monitoring. The objectives of this study were to identify *T. evansi* in blood samples from horses, investigate its occurrence in horses in this region, and analyze the associated risk factors. Three hundred blood samples were collected for parasitological (blood smear), serological (indirect enzyme-linked immunosorbent assay - ELISA), and molecular (polymerase chain reaction - PCR) diagnostic purposes. No trypomastigote forms of *T. evansi* were observed in the examined blood smears. Serological examined of 209 samples revealed that 33.01% (69/209) were positive for anti-*T. evansi* antibodies. No variable was identified as a risk factor. Among the 300 samples submitted to PCR, 1% (3/300) were positive, and sequencing showed 100% similarity with *T. evansi* species. The study identified exposure and infection of horses by *T. evansi* in Maranhão, expanding its geographic distribution in the country and highlighting the importance of periodic testing.

## Resumo

O hemoprotozoário *Trypanosoma evansi* é um parasito que infecta mamíferos, desencadeando uma infecção chamada tripanosomose. No estado do Maranhão, nordeste do Brasil, não há relatos de *T. evansi* em cavalos, destacando a necessidade de avaliar a exposição e infecção pelo parasita e gerar dados para seu monitoramento. Os objetivos deste estudo foram identificar *T. evansi* em amostras sanguíneas de equinos, investigar sua ocorrência em cavalos dessa região e analisar os fatores de risco associados. Foram coletadas 300 amostras de sangue para fins de diagnóstico parasitológico (esfregaço sanguíneo), sorológico (ensaio imunoenzimático indireto - ELISA) e molecular (reação em cadeia pela polimerase - PCR). Nenhuma forma tripomastigota de *T. evansi* foi observada nos esfregaços sanguíneos examinados. A análise sorológica em 209 amostras detectou que 33,01% (69/209) apresentaram anticorpos anti-*T. evansi*. Nenhuma variável foi identificada como fator de risco. Entre as 300 amostras submetidas à PCR, 1% (3/300) apresentaram resultado positivo e, após sequenciamento, revelaram 100% de similaridade com amostras de *T. evansi*. O estudo revelou a exposição e infecção de cavalos por *T. evansi* no Maranhão, expandindo sua distribuição geográfica no país e destacando a importância de testes periódicos

## Introduction

The flagellated hemoprotozoan species *Trypanosoma (Trypanozoon) evansi* (Steel, 1885) belongs to the family Trypanosomatidae (Hoare, 1972). *T. evansi* is the etiological agent of a debilitating disease that can affect wild and domestic mammals. This disease is commonly known as “*surra*” in Asian and African countries, and as “*quebra bunda*,” “*derrengadera*” and “*mal de cadeiras*,” in Brazil (Hoare, 1972; Desquesnes et al., 2013; Jaimes-Dueñez et al., 2017).

The epidemiology of *T. evansi* in equines is influenced by various environmental, geographical and management factors, which determine the spread and transmission dynamics of the parasite (Kasozi et al., 2021; Desquesnes et al., 2022). One of the key transmission mechanisms is mechanical transmission by hematophagous insects, particularly flies of the families Tabanidae and Muscidae (Hoare, 1972), including flies of the genera *Stomoxys* and *Tabanus*. This transmission mechanism enables *T. evansi* to spread beyond areas inhabited by tsetse flies (Lun & Desser, 1995; Desquesnes et al., 2013). In Latin America, transmission of trypanosomiasis can also occur through hematophagous bat species, particularly *Desmodus rotundus*, during their feeding (Hoare, 1965). Additionally, oral, vertical, horizontal and iatrogenic transmission routes have been reported on the continent (Sinha et al., 1971; Ogwu & Nuru, 1981; Raina et al., 1985; Desquesnes, 2004; Desquesnes et al., 2013; Narnaware et al., 2016).

The diversity of hosts enables *T. evansi* to spread successfully on a global scale (Kim et al., 2023). This broad host range, including equines, camels, buffaloes and wild animals such as marsupials and rodents, complicates the epidemiology of *T. evansi* by expanding the potential reservoir pool for the parasite (Singh et al., 2016; Narnaware et al., 2016; Fagundes-Moreira et al., 2024). Human infections caused by this parasite have been reported in India (Joshi et al., 2005; Powar et al., 2006; Shegokar et al., 2006) and Asia (Van Vinh Chau et al., 2016), although so far, no cases have been documented in Brazil.

In Brazil, *T. evansi* affects a wide range of animals, leading to high morbidity and mortality rates, particularly among horses and dogs (Herrera et al., 2004). The epidemiological status of trypanosomosis varies across regions, with cases reported in Brazil’s center-west (Dávila et al., 2003), southern (Zanette et al., 2008; Golombieski et al., 2023), and northern regions (Silva et al., 2016). The disease is endemic in the Pantanal region, affecting horses, dogs, cattle, buffalo, capybaras and coatis, and continues to cause losses over time (Nunes & Oshiro, 1990; Dávila et al., 1999; Herrera et al., 2004; Moraes et al., 2007; Zanette et al., 2008; Silva et al., 2010). Marajó Island, where *T. evansi* was first reported in Brazil in 1885 (Jansen, 1941), still experiences trypanosomosis outbreaks, with significant cases recorded in 2011 and 2012 (Silva et al., 2016).

Despite the widespread distribution and impact of *T. evansi*, the attention given to the epidemiology and control of equine trypanosomosis is scant when compared to trypanosomosis among other animals, despite its significant socioeconomic impact and the damage it causes to animal productivity in endemic areas (Birhanu et al., 2015). This lack of focus extends to awareness, control measures, interventions, and research aimed at improving prevention tools. As a result, the epidemiology of the disease remains poorly understood in many regions, particularly in countries where horses have historical, social or economic significance, such as those in South America (Lima & Cintra, 2016). The insufficient attention to the disease exacerbates its impact, leaving many areas, including parts of Brazil, vulnerable to continued outbreaks and economic losses.

Little is known about *T. evansi* infection in horses in northeastern Brazil, particularly in Maranhão, a state located in the transition zone between the Cerrado and Amazon biomes. The paucity of data about its presence in this region underscores the urgent need for regional studies to assess the extent of infection and associated risk factors. This study aims to fill this gap by using parasitological, serological and molecular methods to identify and report the occurrence of *T. evansi* in horses from Maranhão. Understanding the epidemiology of this disease in such a unique ecological context is critical for improving control measures and reducing the socioeconomic impact of the disease on local communities that are dependent on equines.

## Material and Methods

### Study site

The state of Maranhão covers an area of 329,651.496 km^2^ and is located in an Amazon-Cerrado transition zone (Silva et al., 2019; IBGE, 2022). The state has an estimated horse herd of 257,423 animals (IBGE, 2022), which are used for transportation, leisure and sports.

**Figure 1:**
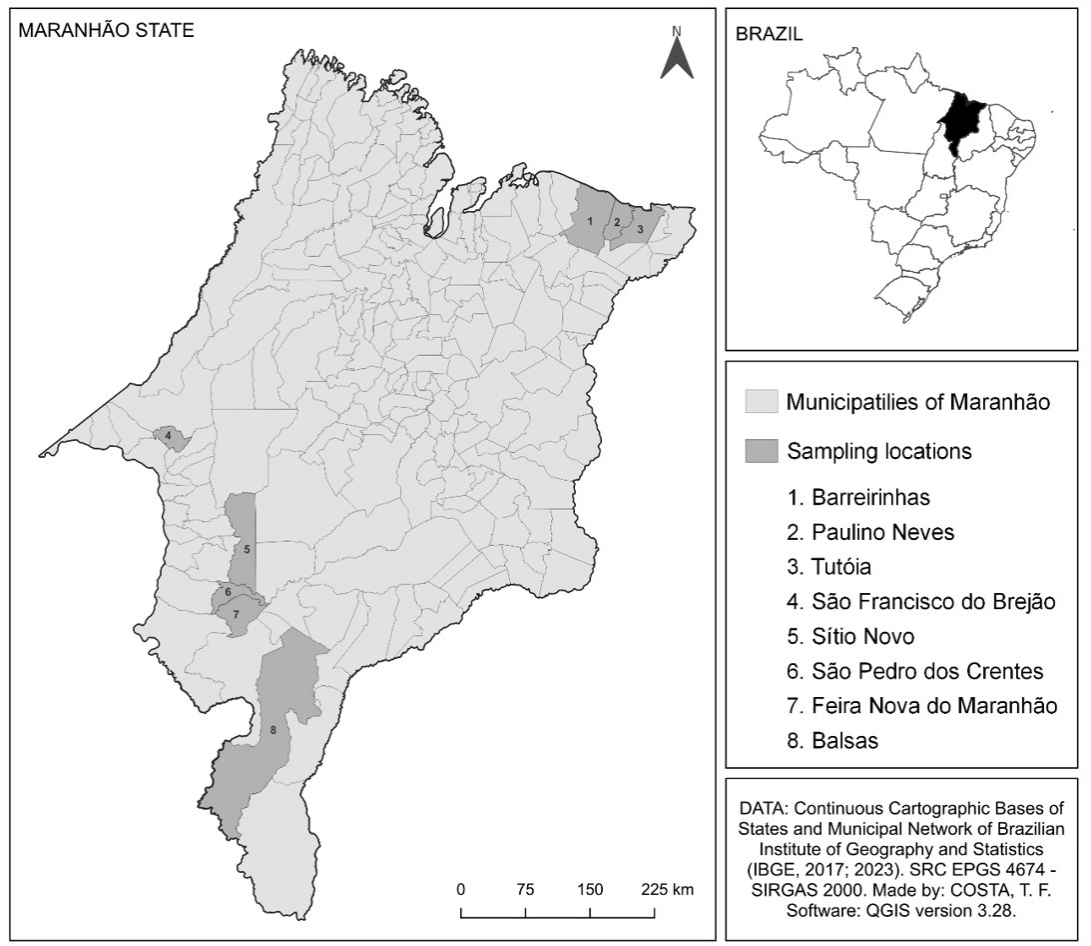
Map showing the municipalities in Maranhão State, Brazil, where the presence of *Trypanosoma evansi* was investigated in equines.

The study was carried out in eight municipalities in the state of Maranhão, Northeastern Brazil (Figure 1), namely, São Francisco do Brejão (latitude: 5° 7' 24” S, longitude: 47° 25' 6” W), Sítio Novo (latitude: 4° 59' 13” S, longitude: 46° 34' 39” W), Barreirinhas (latitude: 2° 44' 58” S, longitude: 42° 49' 58” W), Paulino Neves (latitude: 2° 43' 37” S, longitude: 42° 32' 14” W), Tutóia (latitude: 2° 45' 35” S, longitude: 42° 16' 28” W), São Pedro dos Crentes (latitude: 6° 49' 29” S, longitude: 46° 31' 56” W), Feira Nova do Maranhão (latitude: 6° 57' 16” S, longitude: 46° 40' 44” W) and Balsas (latitude: 7° 31' 59” S, longitude: 46° 2' 6” W).

### Sampling

A cross-sectional observational study was conducted. Sample size was defined based on convenience and the farms involved in the study were selected based on breeders’ willingness to authorize the collection of biological material. Thus, horses aged 1 to 20 years were sampled, regardless of breed, sex, physiological condition, aptitude, and absence or presence of symptomatology. Each animal was considered an independent event, even when two or more animals belonged to the same breeder.

### Epidemiological questionnaire

A semi-structured questionnaire about animal issues and farm management procedures was applied to investigate likely factors associated with equine infection by *T. evansi*.

The person in charge of each farm was asked to fill out an individual data sheet containing information about sex, age and number of animals per farm. Variables such as age and number of animals per farm were subsequently classified as dichotomous. The variable of ‘age’ was classified as (i) over 2 years old, and (ii) equal to or younger than 2 years old. The variable of ‘number of animals per farm’ was classified as (i) one, and (ii) more than one animal raised on the same farm. The classification of ‘number of animals per farm’ aimed to assess whether farm management practices, influenced by animal density, could affect the occurrence of *T. evansi* infection.

### Blood collection and direct parasitological examination

A total of 8 mL of blood was drawn from the jugular vein. Of this total, 4 mL was placed in a tube containing EDTA K_3_ (4 mL, 13 × 75 mm; Firstlab®, FL5-1304S, Ref: 33009, São Paulo, Brazil) for DNA extraction and stored in a cool box for transportation to the laboratory, where it was preserved at –20 °C until further analysis. For serological testing, the remaining 4 mL of blood was placed in a serum separator tube with Serum samples were collected in vacuum tubes containing clot activator and gel separator (5 mL, 13 × 75 mm, yellow cap; Vacuette®, Greiner Bio-One, Brazil). After centrifugation at 2000 rpm for 15 minutes, the serum was aliquoted and stored at –20 °C (Hegazy et al., 2017).

For the morphological examination, one drop of blood from each sample was used to prepare thin-layer blood smears in triplicate at the time of collection. The smears were fixed with methanol for 1 minute, stained with Giemsa, and examined under a light microscope at 40x and 100x magnification with oil immersion, to detect *Trypanosoma* sp. trypomastigote forms, as described by Soulsby (1982), John et al. (1992) and Tehseen et al. (2017).

The health of each horse was assessed by at least two veterinarians (JAS and LMM) during sampling. Both veterinarians confirmed that the horses displayed normal behavior (no prostration, locomotor or neurological disorders) and showed no external signs of clinical disease (e.g., skin conditions, fractures).

### Serological diagnosis

An enzyme-linked immunosorbent assay (ELISA) was performed to assess the animals’ exposure to *T. evansi*, based on the method described by Machado et al. (1997) and Aquino et al. (2010), with modifications. Nunc Maxisorp background plates (Thermo Fisher Scientific, USA) were sensitized with 10 μg/ml (1 μg per well) crude *T. evansi* antigen and blocked with PBS containing Tween 80 and 5% skimmed milk powder. Antigens were obtained from a *T. evansi* strain originally isolated from a naturally infected dog in the Pantanal region of Nhecolândia, and the soluble antigen used in the ELISA assays was prepared according to the protocol described by Aquino et al. (1999). The protein concentration of the soluble antigen was determined using the bicinchoninic acid method (BCA Kit – Pierce Chemical Company).

Control and test sera were diluted in PBS containing Tween 80 at a 1:100 ratio. Rabbit serum containing anti-equine IgG was used as the conjugate, in association with alkaline phosphatase (AB6063, Sigma-Aldrich) at a 1:30,000 dilution. The optical density (OD) results were read on an iMark™ Microplate Absorbance Reader 20052 (Bio-Rad), using a 405 nm filter and phosphatase as the reagent. Two positive controls and two negative controls were added to each plate. OD values observed for negative controls in all plates were used to set the cut-off point based on the following equation described by Gomes et al. (2008): Cut-off point = Mean value of negative controls x 2.5.

### Molecular diagnosis

DNA extraction was performed using the protocol of the DNA Bio Gene Extraction Kit (Quibasa-Bioclin), according to the manufacturer’s recommendations. To assess the quality of extraction, DNA integrity and/or the presence of inhibitors after DNA extraction, the samples were subjected to polymerase chain reaction (PCR) for amplification of the endogenous Cytochrome B gene using oligonucleotides and reactions previously described by Steuber et al. (2005).

A molecular characterization was performed by applying the conventional polymerase chain reaction (PCR) technique, using a Promega GoTaq® Master Mix Commercial Kit. The oligonucleotide primers TBR1: 5’-GAATATTAAACAATGCGCAG-3’ and TBR2: 5’-CCATTTATTAGCTTTGTTGC-3’, described by Masiga et al. (1992), previously proposed to amplify samples with *T. brucei*, were employed to amplify a 164 bp product from the kDNA minicircle region. The reaction conditions, adapted from Masiga et al. (1992), included initial denaturation at 95 °C for 5 min, followed by 35 cycles of denaturation at 95 °C for 1 min, annealing at 49 °C for 1 min, and extension at 72 °C for 10 s, with a final extension at 72 °C for 4 min.

Ultrapure water was used as a negative control, while genomic DNA extracted from the blood of a horse naturally infected with *T. evansi* (previously identified and confirmed by sequencing) served as the positive control.

The PCR products were subjected to horizontal electrophoresis on a 2% agarose gel, stained with SYBR Safe (Invitrogen, USA), and visualized using a blue-light transilluminator (Loccus, Brazil). PCR positive samples were purified using a Wizard Genomic DNA Purification Kit (Promega) and sequenced on an automated sequencer (ABI-PRISM 3500 Genetic Analyzer, Foster City, USA).

Using the BLASTn algorithm, the sequences obtained were compared with the nr/nt database at NCBI to confirm species identity based on their similarity to sequences available in GenBank. The comparison was based on the e-value score, with significant matches considered indicative of high confidence in the sequence identities.

### Statistical analysis

The statistical processing software Epi Info™, version 7.2.4 (CDC, United States), was used to determine the frequency of *T. evansi*-positive animals in PCR and serology analyses. The correlation between seropositive animals and the variables of ‘sex,’ ‘age’ and ‘number of animals per farm’ was investigated via the Mantel-Haenszel chi-square correlation test, based on a two-tailed test conducted for a p-value of < 0.05. The following relative risks were used as association parameters: relative risk < 1 featured protective factor, and relative risk > 1 was the risk factor. The relative risk value was subtracted from 1 to find the relative risk rate.

## Results

A total of 300 horses were sampled, distributed as follows: São Francisco do Brejão, n = 54; Sítio Novo, n = 40; Balsas, n = 97; Barreirinhas, n = 25; Feira Nova do Maranhão, n = 6; Paulino Neves, n = 61; São Pedro dos Crentes; n = 13; Tutóia, n=4.

Of the 300 horses assessed, 192 (64%) were males and 108 (36%) were females. The age of the horses ranged from 1 to 20 years, with a mean age of 5 years. For improved analysis, the horses were grouped into the following age ranges: 1-2 years, 120 horses (40%); 3-9 years, 156 horses (52%); and 10-20 years, 24 horses (8%). The majority of the horses (92%) were under 10 years old, which reflects the predominantly young population in the study.

Furthermore, an analysis of the distribution of farms showed that in 60% (72/120) of the farms visited, at least two horses were kept together, revealing that the majority of them kept more than one horse.

Regarding the parasitological diagnosis for the detection of *T. evansi* trypomastigotes in blood smears, none of the examined slides tested positive indicating a low parasitemia, and consequently a greater difficulty in finding it in the blood smear test.

However, the serological tests of 209 blood samples detected that 33.01% (69/209) of the animals tested positive for *T. evansi*, indicating prior exposure to the parasite. No statistical differences were found between seropositive animals and the evaluated variables, including sex, age, and number of animals per farm. The analysis did not identify any of these factors as either protective or risk factors for *T. evansi* infection in the horse population under study.

Using the PCR test targeting the kinetoplast minicircle DNA gene with TBR-1/TBR-2 oligonucleotides, 1% (3/300) of the samples tested positive for *T. evansi*. The positive samples presented the expected band of approximately 164 bp in agarose gel. Genetic sequencing and similarity analysis with GenBank sequences revealed 100% identity with *T. evansi* species isolated from camels in Sudan (MF142291.1) and Pakistan (OQ174990.1). The sequences obtained in this study were deposited in GenBank under accession numbers PQ425574 and PQ373893.

In a comparative analysis of PCR and ELISA testing (Table 1), we observed the following: of the three PCR-positive samples, one was also positive in the ELISA test, another yielded a negative ELISA result, and for the third, ELISA testing was not possible due to insufficient serum.

**Table 1:** Results of serological and molecular diagnosis of *Trypanosoma evansi* in horses, Maranhão State, Brazil, with samples collected in 2022 and 2023.

**Municipality**	**ELISA**	**PCR**
	**% (n/N)**	**% (n/N)**
Paulino Neves *	-	1.63 (1/61)
Barreirinhas	31.81 (7/22)	0.0 (0/25)
Tutoia *	-	0.0 (0/4)
Balsas	29.33 (22/75)	2.06 (2/97)
Feira Nova do Maranhão	16.66 (1/6)	0.0 (0/6)
São Pedro dos Crentes	46.15 (6/13)	0.0 (0/13)
São Francisco do Brejão	43.39 (23/53)	0.0 (0/54)
Sitio Novo	25 (10/40)	0.0 (0/40)
**Total**	**33.01 (69/209)**	**1 (3/300)**

PCR: Polymerase Chain Reaction; % = percentage; n= number of positive cases; N= number of tested samples.

* ELISA test was not performed.

Among the PCR-positive samples, two were from male horses raised in Balsas. Both animals, approximately two years old, were raised on the same farm. The third sample came from a 5-year-old male horse raised in Paulino Neves, and it was the only animal from that farm.

## Discussion

Horses are widely used in northeastern Brazil for livestock farming, leisure, cultural events, sports and ecotourism. They are frequently purchased and transported between municipalities and states for these purposes, a practice that is among the most common and efficient pathways for introducing diseases, such as trypanosomosis, into previously disease-free environments (Tamarit et al., 2011).

Infections caused by *T. evansi* in horses have been reported in Brazil’s central-west (Dávila et al., 2003), southern (Zanette et al., 2008; Golombieski et al., 2023), northern (Silva et al., 2016), and southeastern (Nunes et al., 2012) regions. The results of this study provide the first evidence of this parasite in northeastern Brazil, in contrast to a previous survey in the state of Bahia, where no positive cases were found (Costa et al., 2019).

The detection of *T. evansi* in this study, however, was not corroborated by the identification of forms suggestive of trypomastigotes in the blood smear exams. This limitation may be related to the technique itself, since blood smears typically show good sensitivity only in cases of high parasite loads (Woo, 1970). Low parasite loads make it nearly impossible to detect and morphologically differentiate the parasite using Giemsa-stained blood smears (Ramírez-Iglesias et al., 2016).

It is well known that *T. evansi*-infected horses can have subclinical infections without presenting obvious clinical signs, often resulting in low parasitemia that further complicates detection (Büscher et al., 2019). The absence of trypomastigotes in the analyzed samples may be explained by the fact that the animals were not in the active phase of infection, when parasites circulate in large blood vessels. Such a low parasite load may also indicate the possibility that the animals were in the chronic phase of the disease. During this phase, the host’s immune system partially controls the infection, leading to a reduced number of blood-circulating parasites and hindering their detection through traditional parasitological methods (Shahzad et al., 2010; Al Malki & Hussien, 2022). These results highlight the limitations of blood smear analysis and confirm the superiority of PCR as a diagnostic tool for the detection of trypanosomosis in cattle.

The frequency of *T. evansi* sero-reactive animals found in the present study (33.01%) is similar to that found in the southern region of the Americas. This parasite is widely distributed in Latin America and endemic to subtropical Argentinean (Monzón et al., 1995) and Paraguayan (Suganuma et al., 2022) regions. However, in Brazil, a lower seroprevalence rate of 9.6% (31/321) was reported in the state of Mato Grosso, Center-West (Herrera et al., 2004). These differences may be influenced by ecological and epidemiological factors, such as variations in vector density, wildlife reservoirs, management practices, and environmental conditions that affect parasite transmission dynamics.

The ELISA test allows for the rapid and cost-effective analysis of large sample sets, detecting antibodies and identifying animals exposed to the parasite, even after its elimination. While ELISA often yields higher positive results due to its ability to reveal past infections or carriers, its limitations include cross-reactivity with antibodies from other infections and the persistence of antibodies post-treatment, complicating the differentiation between active and resolved infections. PCR complements ELISA by identifying parasite DNA, confirming active infections with high specificity, particularly in endemic areas with low parasite loads or subclinical cases. Molecular tests detect parasite-specific DNA or RNA with high accuracy, and these markers disappear shortly after successful treatment, making PCR a valuable tool for diagnosing recent infections (Njiru et al., 2006; Ramírez-Iglesias et al., 2016; Benfodil et al., 2020).

In line with the above, the frequency of *T. evansi* infection observed in the current study through PCR (1%). In contrast, higher prevalence rates have been reported in different regions of Brazil, such as 8.2% (20/243) in the state of Pará, North (Silva et al., 2016); 33.3% (2/6) in the state of Minas Gerais, Southeast (Nunes et al., 2012); and 34.9% (112/321) in the state of Mato Grosso, Center-West (Herrera et al., 2004).

Although the aforementioned study reported a low frequency of animals positive for TBR-1/TBR-2 oligonucleotides belonging to the mitochondrial minicircle gene (kDNA), one cannot rule out higher frequencies, since these oligonucleotides have their limitations when it comes to chronic infections with parasitic loads below 10 fg of parasite DNA (Herrera et al., 2004). Likewise, the incidence of weak bands in the DNA amplification process may have resulted from low a parasite load, as described by Shyam et al. (2013). Benfodil et al. (2020) recommend combining serological and molecular tests to ensure the accurate diagnosis of *T. evansi* infection in domestic animals, given the persistence of circulating antibodies detectable by serological tests in cured animals.

Although serological tests such as ELISA can be used effectively to determine the prevalence of *T. evansi* infection (Reck et al., 2021; Desquesnes et al., 2022), PCR has been highlighted by Ahmed et al. (2013) as the most reliable method for investigating the incidence of this infection due to the specificity and ability of PCR to confirm active infections. The combined use of ELISA and PCR is therefore essential in epidemiological studies, because it enhances the accuracy of infection detection and monitoring, enabling more robust surveillance and control of *T. evansi*.

The epidemiology of equine trypanosomosis caused by *T. evansi* in Latin America requires the presence of a mechanical vector, in addition to that of a susceptible and a carrier animal. Thus, the diverse and abundant tabanid fauna in the state of Maranhão comprises species of the genus *Tabanus,* which are widely distributed in several regions in the state (Limeira-de-Oliveira, 2003) and have already been associated with the transmission of the investigated parasite (Baldacchino et al. 2014). The presence of these insects is an important factor favoring both the incidence and dissemination of this parasite in the investigated region. Most importantly, although climate change affects the distribution of dipteran species, consequences associated with vector-transmitted parasitic diseases remain little known. Moreover, there is a paucity of knowledge about the consequences of pathogen-vector species such as *Lepiselaga crassipes* (Diptera: Tabanidae) for production animals, since this species acts as a mechanical vector of trypanosomosis, affecting horses and cattle in the Neotropical region (Marques et al., 2017).

The number of common house flies and horse flies in hot and humid regions increases considerably during the rainy season, which occurs between January and June in the region under study here (Carvalho, 2020). Therefore, this is the most likely season when the highest agent-transmission rates for diseases transmitted by these etiological vectors are recorded (Silva et al., 2016). According to horse owners, these insects make their animals very restless, causing them to reduce their intake of food and water. Consequently, their nutrition is compromised and their immunity declines, favoring the onset of infections by several other etiological agents.

Although no statistically significant association was found between the number of animals per farm, sex and age, horses raised in the same environment favored transmission by pathogen vectors, particularly through mechanical transmission (Foil, 1989; Rodrigues et al., 2005). This finding is substantiated by the fact that vector flies and horse flies do not travel long distances to feed on blood. On the contrary, these insects have the habit of obtaining the blood they need for their egg-laying process in the same place where they live. By so doing, they save the energy needed to travel long distances. Thus, they limit their displacements to less than one kilometer (Desquesnes et al., 2013).

It should be noted that a statistical comparison between the dry and rainy seasons was not performed in this study, since most of the samples were collected during the dry season due to limited access to farms in the rainy season. Therefore, the unequal distribution of samples across the periods precluded a valid comparative analysis. Furthermore, this study did not include an investigation of vectors and treatments, since these factors fell outside the original scope of the investigation, which aimed primarily at identifying the occurrence of *T. evansi* in horses. Although the analysis of vectors and treatments is relevant, these variables may be explored in more detail in future research.

The seroreactive animals showed no clinical signs of trypanosomosis, but, according to Elhaig & Sallam (2018), *T. evansi* causes acute infection in camels, horses and dogs, which is manifested through fever, weight loss, anemia, genital inflammation, neurological symptoms, and sometimes death. Therefore, it is worth emphasizing the importance of conducting the early diagnosis of *T. evansi* infection so that sanitary measures aimed at preventing major losses can be taken should this disease spread in the horse herd.

The findings of this study emphasize the critical need for robust monitoring and control strategies for *T. evansi* in equine populations, particularly in regions where the disease remains underreported. The identification of *T. evansi* in the area of this study underscores the vulnerability of equids to this protozoan parasite, which can lead to severe health consequences in horses. Furthermore, the parasite poses a significant risk to the broader ecosystem, as it can be transmitted to other animal species, including cattle (bovines and buffaloes) and wild animals (Singh et al., 2016; Narnaware et al., 2016; Fagundes-Moreira et al., 2024). This parasite also has zoonotic potential, since humans may become infected (Joshi et al., 2005; Powar et al., 2006; Shegokar et al., 2006; Van Vinh Chau et al., 2016) through contact with vectors such as *Tabanidae* flies, which facilitate parasite transmission.

The absence of a comprehensive control program in many parts of Brazil highlights the need for a coordinated effort among animal health protection agencies, producers, and technical managers to address gaps in disease surveillance, diagnostic capabilities, and prevention strategies. In view of this fact, we emphasize the importance of continued research for the development of more effective diagnostic methods, treatment options, and potential vaccines against *T. evansi*.

Trypanosomiasis has a significant economic impact on livestock farming, especially in rural and economically disadvantaged areas. Effective control measures can directly benefit producers by improving animal welfare and reducing financial losses. In this context, the findings of this study represent an important step toward a better understanding of the distribution of *T. evansi* in northeastern Brazil and highlight the need for coordinated and sustained efforts in the monitoring, prevention, and control of the disease to protect animal.

## Conclusions

The detection of *T. evansi* in horses in the state of Maranhão is a significant finding, and this is the first report of its occurrence in this region. These results expand the known geographical distribution of *T. evansi* and underscore the need for further epidemiological studies to understand its impact on animal health. This study emphasizes the importance of monitoring and controlling trypanosomosis in areas within the Cerrado-Amazon transition zone to mitigate its effects on equine populations and safeguard the health of animals.

## Data Availability

The data that support the findings of this study are available from the corresponding author upon reasonable request.
